# PSO-BP Neural Network-Based Strain Prediction of Wind Turbine Blades

**DOI:** 10.3390/ma12121889

**Published:** 2019-06-12

**Authors:** Xin Liu, Zheng Liu, Zhongwei Liang, Shun-Peng Zhu, José A. F. O. Correia, Abílio M. P. De Jesus

**Affiliations:** 1 Department of industrial product design, Guangzhou University, Guangzhou 510006, China; designer_liuxin@163.com; 2 School of Mechanical and Electrical Engineering, Guangzhou University, Guangzhou 510006, China; lzwstalin@126.com; 3 School of Mechanical and Electrical Engineering, University of Electronic Science and Technology of China, Chengdu 611731, China; zspeng2007@uestc.edu.cn; 4 INEGI, Faculty of Engineering, University of Porto, 4200-465 Porto, Portugal; ajesus@fe.up.pt

**Keywords:** wind turbine blade, full-scale static test, PSO-BP Neural Network, strain prediction

## Abstract

The full-scale static testing of wind turbine blades is an effective means to verify the accuracy and rationality of the blade design, and it is an indispensable part in the blade certification process. In the full-scale static experiments, the strain of the wind turbine blade is related to the applied loads, loading positions, stiffness, deflection, and other factors. At present, researches focus on the analysis of blade failure causes, blade load-bearing capacity, and parameter measurement methods in addition to the correlation analysis between the strain and the applied loads primarily. However, they neglect the loading positions and blade displacements. The correlation among the strain and applied loads, loading positions, displacements, etc. is nonlinear; besides that, the number of design variables is numerous, and thus the calculation and prediction of the blade strain are quite complicated and difficult using traditional numerical methods. Moreover, in full-scale static testing, the number of measuring points and strain gauges are limited, so the test data have insufficient significance to the calibration of the blade design. This paper has performed a study on the new strain prediction method by introducing intelligent algorithms. Back propagation neural network (BPNN) improved by Particle Swarm Optimization (PSO) has significant advantages in dealing with non-linear fitting and multi-input parameters. Models based on BPNN improved by PSO (PSO-BPNN) have better robustness and accuracy. Based on the advantages of the neural network in dealing with complex problems, a strain-predictive PSO-BPNN model for full-scale static experiment of a certain wind turbine blade was established. In addition, the strain values for the unmeasured points were predicted. The accuracy of the PSO-BPNN prediction model was verified by comparing with the BPNN model and the simulation test. Both the applicability and usability of strain-predictive neural network models were verified by comparing the prediction results with simulation outcomes. The comparison results show that PSO-BPNN can be utilized to predict the strain of unmeasured points of wind turbine blades during static testing, and this provides more data for characteristic structural parameters calculation.

## 1. Introduction

Both the reliability and stability of wind turbine blades affect the safety of the whole machine directly. In order to check the rationality of blade design and verify the safety of manufacturing, static experiments of prototype blades have been performed as a necessary part of the blade certification process [1]. Through static experiments of wind turbine blades, the verification of the designed loading capacity of the blades can be built, and the information about structural characteristics, strain and deformation under the test load can be obtained [2]. Existing literature reports substantial researches on the structural testing of wind turbine blades. For example, Jensen et al. [3] continuously loaded a 34-m-long blade in the flap-direction until the blade failed, recorded the displacements throughout the loading history by local displacement measurement equipment, and found that the peeling of the skin and the box girder were the main cause of blade instability. Through the full-scale fatigue experiment of a 3 MW wind turbine blade directed by IEC 61400-23, Lee et al. [4] found that delamination failure will happen at the blade root and figured out the causes of the delamination failure and problems of the conventional design approach by simulating the situations experienced by the blade. During the conditional monitoring on the trailing edge in a full-scale fatigue experiment of a 2 MW wind turbine blade, Pan et al. [5] found that the stress concentration will lead to delamination between GFRP and the balsa wood, and then proposed a method to increase the core materials in the trailing edge by computing the local stress distribution and stability factors, based on finite element calculations. Lee and Park [6] carried out static testing on a 48.3-m-long blade which had initial static testing and fatigue testing. They found that the blade collapsed when the applied load surpassed 70% of the target value. In addition, Lee and Park [6] proposed an improved laminate pattern to enhance the residual strength of the wind turbine blade.

The references as above are mainly related to the analysis of the failure modes as well as failure causes of wind turbine blades. Moreover, measurement and calculation methods of structural characteristic parameters have also been intensively studied, including the influence of defects and size effect [7,8,9]. Based on the method of finite differences and an arbitrary beam bending and moment theory, Choi et al. [10] proposed a tip deflection calculation method based on the measured strains data analysis for wind turbine blades, and in order to verify the proposed method, they conducted static testing on a 100 kW wind turbine blade with FBG sensors embedded in its shear web; the average calculation error of the proposed method was proved to be within 2.25%. Before performing a static experiment with a 100 kW wind turbine blade, Kim et al. [11] installed FBG sensors into the bonding line among the shear web and spar cap to collect the strain data and then they found that the collected strain data can be effectively used to evaluate the deflections of the wind turbine blades. Roczek-Sieradzan et al. [12] preformed a full-scale static test of a certain wind turbine blade under the combined loadings of flag and edgewise directions, with the overall and local deformation information measured and recorded during the experimental process and proved that the measurement results can be effectively used to analyze the structural performance of wind turbine blades. On the basis of the digital image analysis technology, Dou [13] proposed a deformation testing method for the full-scale static experiment for wind turbine blades and completed the data measurements and analysis of the three-dimensional deformation field. Shi et al. [14] studied the impact of time-varying environmental temperature and humidity on the test results on the basis of a fatigue experiment of a 1.5 MW wind turbine blade; this research could provide a basis for the development and structure testing of wind turbine blades. Pan [15] studied the influence of structure nonlinearity on the experimental results of full-scale wind turbine blades static testing, analyzing the relationship among bending moment, strain, stiffness, and deflection, among others, and provided a more accurate stiffness data for numerical loading calculations. Yan et al. [16] tested and recorded the frequency, deflection and strain of a 48-m-long wind turbine blade, compared the test results with the design value and found that the error range satisfied the DNV-GL2015 specifications.

The above-mentioned researches have made significant achievements in the analysis of blade failure causes, blade load-bearing capacity, and the measurement methods of the full-scale blade static testing, and there are also some researchers who have investigated the blade structural characteristics analysis from the physics of failure mechanisms [17,18,19,20,21,22]. However, studies on the correlation analysis of the strain, the applied loads, the loading positions, and displacements in static testing are scarce. Actually, in the static testing, the relationships of the strain with the applied loads, loading positions, displacements, etc., are nonlinear, and the number of design variables is numerous [15,23], thus the calculation and prediction of blade structural characteristics are very complicated. Moreover, the number of measuring points and strain gauges in full-scale wind turbine blade static testing are usually limited, thus the structural characteristics of unmeasured points cannot be directly obtained, so that the static testing has little significance for the calibration of blade design [15]. Considering this problem, the methods of Particle Swarm Optimization (PSO) and Neural Networks are considered in this paper. Actually, there are a few studies regarding Particle Swarm Optimization (PSO) and Neural Networks applied to the wind turbine blades analysis. Andrew Kusiak [24] proposed that Neural Networks improved by PSO were applied in the adaptive control of a wind turbine. Cynthia [25] introduced PSO and Neural Networks optimization methodology to optimize the wind velocity and attack angle of a horizontal axis wind turbine in order to obtain the maximum power coefficient. Milad Fooladi [26] applied Neural Networks improved by PSO to assess the different factors affecting flicker in wind turbines. So, the Neural Networks improved by PSO used to solve the problems of wind turbine blades is effective and efficient. However, there are fewer researches about PSO-BPNN in the wind turbine blades studies. Wang Lei [27] applied PSO-BPNN to perform a structural analysis approximation of wind turbine blades, and the effectiveness of the approach was demonstrated. 

As a result of a literature review and the concerns raised above, this paper aims at presenting a study on new strain prediction methods by introducing intelligent algorithms. As mentioned above, PSO-BPNN has significant advantages in dealing with non-linear fitting and multi-input parameters, and the models constructed by PSO-BPNN have better robustness and accuracy [28]; thus, PSO-BPNN has been introduced to predict the strain of wind turbine blades in this paper and a new strain-predictive PSO-BPNN model for full-scale wind turbine blades static behavior to be established. The new model can be used to predict the strain values of the unmeasured points and provide more strain data for structural characteristic parameters calculation.

The structure of this paper is organized as follows: Section 2 introduces the conditions and test procedures for the full-scale static testing of a wind turbine blade; the basic concepts of Neural Networks as well as PSO-BPNN are introduced in Section 3; in Section 4, the strain-predictive method for the central of pressure side based on PSO-BPNN is studied; Section 5 presents the conclusions of this research.

## 2. Full-Scale Static Testing of a Certain Wind Turbine Blade

### 2.1. The Wind Turbine Blade Introduction

**Figure 1:**
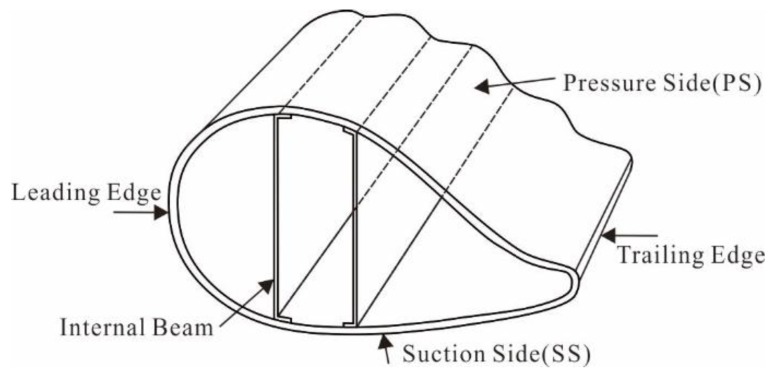
The wind turbine blade structure used in this research.

In this research, a blade produced with a box beam structure for the main girder was used. The matrix material is alkali-free glass fibers impregnated with epoxy resin, and the reinforcing phase material is impregnated glass fibre. The mass of this blade is 15,982 kg with an inherent natural frequency of 1.41 Hz, and the main information regarding the turbine blade is listed in Table 1. In the structure of the wind turbine blade, 0° fibres are applied to increase flap-wise bending stiffness, while ± 45° fibres are applied to increase torsional stiffness. Figure 1 illustrates the structure of the wind turbine blade.

### 2.2. Strain Gauges Arrangement

**Figure 2:**
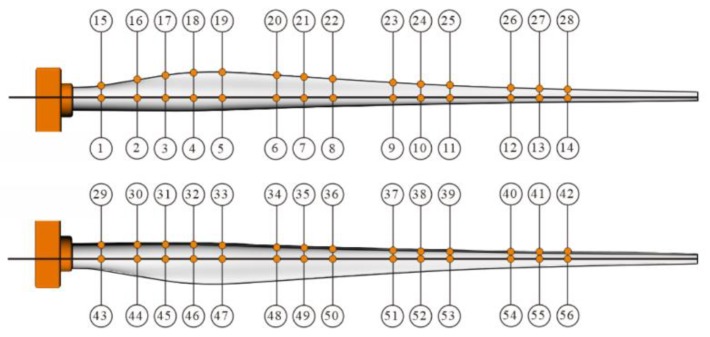
The strain gauges arrangement in the turbine blade.

According to Figure 2, 56 strain gauges were attached to the surface of the wind turbine blade along the pressure side (PS), suction side (SS), and the leading edge in addition with the trailing edge, before static testing.

### 2.3. Testing Process

**Figure 3:**
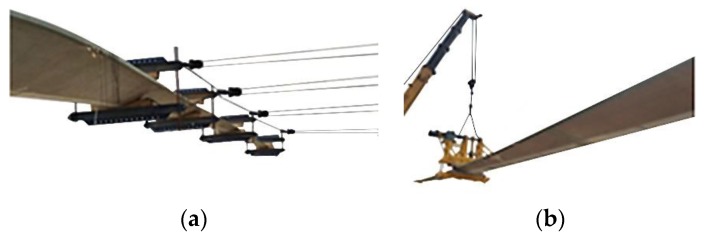
Full-scale static experiments of the wind turbine blade. (**a**) Side pulling in the full-scale static experiment of the wind turbine blade; (**b**) Lifting in the full-scale static experiment of the wind turbine blade.

In this study, two static tests are successively carried out from different directions, edgewise+ and edgewise−, as shown in Figure 3. This paper will establish the strain prediction models to predict the strain values of the unmeasured points on the center of the pressure side in the two static tests.

**Figure 4:**
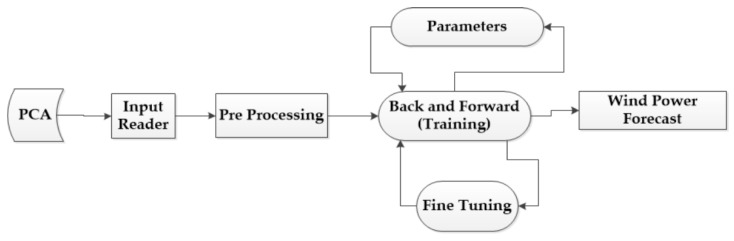
The applied loading diagram of the full-scale static testing.

By means of 64 bolts, the root of the blade is installed on the test platform. For the static testing of edgewise+ and edgewise−, five loading positions were chosen and the loading points arranged according the distances of 18.00 m, 30.00 m, 42.00 m, 50.00 m, and 60.00 m measured from the root of the blade, respectively, as shown in Figure 4. It is worth noting that a crane is used to avoid the blade tip landing in the test of edgewise+ for large deflection. In addition, the limit load is performed by the side pull, and the target load of each loading point is shown in Table 2. For every loading point, the loading direction is perpendicular to the normal direction of the loading part. In Figure 4, P1–P5 are the positions of the tensile machine, P6 is the crane, and S1–S5 are the load application points.

Before starting the test, the applied load, displacement and strain of the blade have been cleared. Then, using the lateral loading device, the blade was loaded by 0%, 40%, 60%, 80%, and 100% of the target load, step by step, while the displacement data and the strain gauge data were recorded during the loading process. After the loading process was completed, the blade was unloaded to the zero state, step by step. The load of each stage on directions of edgewise+ and edgewise− are shown in Table 3 and Table 4.

## 3. BPNN Improved by PSO

### 3.1. The Brief Principles of BPNN

**Figure 5:**
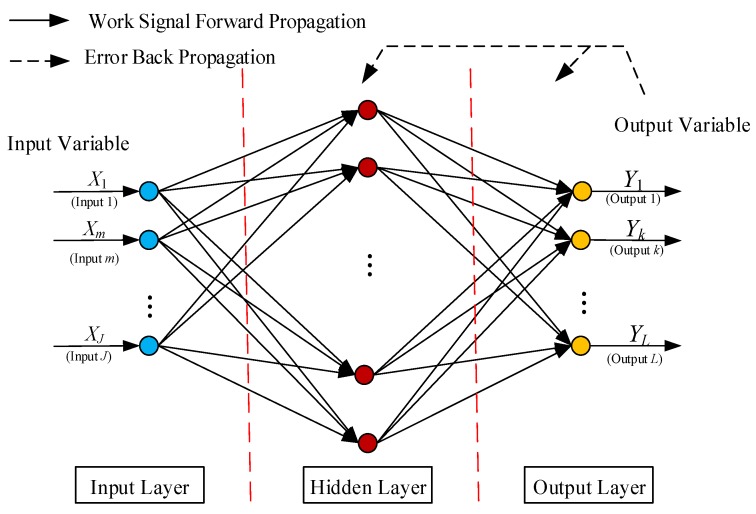
The topology of BPNN.

The feed-forward BPNN with three layers is proposed for predicting the wind turbine blade static test strain. The first layer is the input layer describing the input data such as values in terms of the distances of points measured from the root of the blade, the applied load of each stage in flap and so on. The second layer is the hidden layer comprising Neurons. The third layer is the output layer describing the output variables such as values of the strain predictions. The data are processed through the second and third layer with the activation function [29]. The relationship model between input set $\left\{X_{m}|m=1,2,\cdots ,J\right\}$ and strain Y is established using an improved neural network algorithm. With samples $X_{1},\cdots ,X_{m}$ as the input values, and $Y_{1},\cdots ,Y_{L}$ as the output values, the strain prediction model is trained. The network architecture of BPNN predicting the wind turbine blade static test strain is illustrated by Figure 5.

### 3.2. The BPNN Optimization Principles Based on PSO

**Figure 6:**
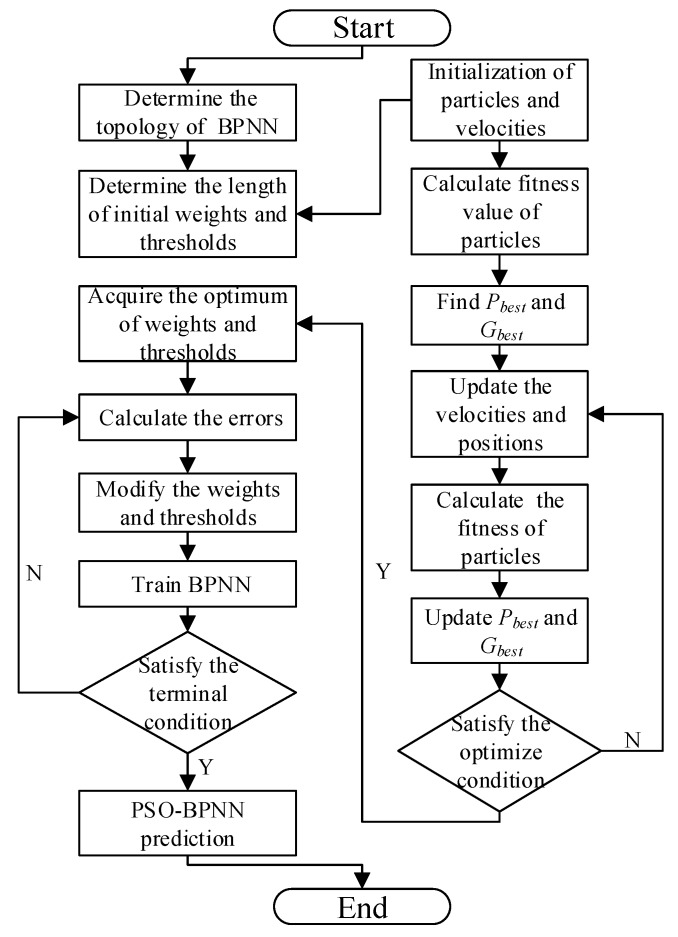
The optimization process of PSO-BPNN.

The traditional BPNN utilizes the gradient descent method generally, so the PSO-BPNN in our research is improved by Levenberg-Marquart. The convergence of BPNN is mainly dependent on the initial weights and thresholds and it is likely to obtain the local minimum. According to these characteristics of the BPNN, it is a kind of popular and effective method to overcome the defects that the naturally inspired algorithms have [30]. In this research, the weight and threshold values of BPNN, which can effectively prevent the training from falling into the local optimal situation, are optimized by PSO, which means errors of this neural network training are minimized. In addition, PSO is easy to use and perform its function because it is not necessary to set parameters for evolving such as crossover operator and mutational operator [31]. The improved BPNN process based on PSO is shown in Figure 6. Many possible initial values can be set by PSO, which updates them in a range of continuous iterations. The different conditional combinations can be achieved in each iteration until the biggest fitness function value is obtained and the output values are computed. The optimization process of PSO-BPNN is shown in Figure 6.

PSO has good global search capability, accordingly updating the velocities and positions of particles, the global best particle is found. The weight and threshold of BPNN are optimized, then the BPNN is trained. After that, the optimization structure of BPNN is completed. The steps of BPNN improved by PSO are summarized as follows [32]:

First of all, the size of the population is set. The frontier $\left[-X_{\max},X_{\max}\right]$, the biggest velocity $\left[-V_{\max},V_{\max}\right]$, inertia weight coefficient w, the maximum iterative number, and the acceleration constants $c_{1}$, $c_{2}$ are set. The position *X_i_* and velocity *V_i_* are initialized.

Second, the fitness values of each particle are calculated. According the fitness function, all the fitness values are obtained. The higher the fitness values are, the better the performance in particles is. Meanwhile, the individual best positions $P_{best}$ and the global optimum positions of particles $G_{best}$ are updated and recorded.

Third, the velocity and position of every particle updated can be obtained using Equations (1) and (2) [33]:

(1) $$V_{ij}^{k+1}=wV_{ij}^{k}+c_{1}r_{1}\left(P_{ij}^{k}-X_{ij}^{k}\right)+c_{2}r_{2}\left(G_{j}^{k}-X_{ij}^{k}\right)$$

(2) $$X_{ij}^{k+1}=X_{ij}^{k}+V_{ij}^{k+1},1\leq i\leq n,1\leq j\leq d$$

where *k* represents discrete time index, w represents inertia weight factor, *c*_1_ and *c*_2_ represent acceleration constants, and $r_{1}$ and $r_{2}$ are uniformly distributed random numbers on the interval [0, 1]. $P_{ij}^{k}$ is dimension *j* of the best point vector found by the *i*-th particle in the *k*-th iteration, and $G_{j}^{k}$ is dimension *j* of the global best point in the *k*-th iteration. $X_{ij}^{k}$ represents dimension *j* of the position vector found by the *i*th particle in the *k*-th iteration, and $V_{ij}^{k+1}$ is dimension *j* of the velocity vector found by the *i*-th particle in the (*k* + 1)-th iteration.

Generally, if the velocities of the flying swarms are not restricted appropriately during the PSO calculation, the swarms may fly into the local optimization, which means the flying particles cannot reach the global optimum. For the purpose of the particles based on the restricted velocity to obtain the optimal solution, *V*_max_ as the velocity threshold is introduced, and the limited conditions are imposed as follows:

$$\mathrm{If}\;V_{ij}{}^{\left(k+1\right)}>V_{\max},\;\mathrm{then}\;V_{ij}{}^{(k+1)}=V_{\max};$$

$$\mathrm{If}\;V_{ij}{}^{\left(k+1\right)}<-V_{\max},\;\mathrm{then}\;V_{ij}{}^{(k+1)}=-V_{\max};\;\mathrm{else}\;V_{ij}{}^{(k+1)}\mathrm{unchanged}.$$

$$\mathrm{If}\;X_{ij}{}^{\left(k+1\right)}>X_{\max},\;\mathrm{then}\;X_{ij}{}^{\left(k+1\right)}=X_{\max};\;\mathrm{If}\;X_{ij}{}^{\left(k+1\right)}<X_{\min},\mathrm{then}\;X_{ij}{}^{(k+1)}=X_{\min}.$$

Fourth, if the algorithm reaches the maximum iteration or the precision of error is smaller than the setting value, the point of the current optimization swarm is outputted. Otherwise, this process should return to the third step and continue to train the PSO model. 

Fifth, $G_{best}$ outputted depending on the fourth step are as the initial inputted values of the weight and threshold of BPNN. In the PSO-BPNN algorithm, every dimension of the vector $x_{i}=(x_{i1},x_{i2},\cdots ,x_{is})$ represents the weight or threshold values of BPNN; *s* is the amount of weights and thresholds in BPNN. The fitness functions of particle swarms are as Formulae (3) and (4) [32]:

(3) $$L_{i}=\sum_{j=1}^{s}(y_{ij}-Y_{ij}{)}^{2}$$

(4) $$L_{popIndex}=\frac{1}{m}\sum_{i=1}^{m}L_{i}$$

where *m* is the number of particles describing population size, $Y_{ij}$ is the ideal outputted value *j* of the *i*-th particle, $Y_{ij}$ is the actual outputted value *j* of the *i*-th particle; popIndex=1,2,⋯,m. $L_{popIndex}$ is the fitness of particle *popIndex*.

Sixth, the established training set is applied to the optimized BPNN algorithm. The transfer function is the tangent S-type transfer function between the input layer and the hidden layer too, and the transfer function is also set as a linear transfer function between the hidden layer and the output layer.

## 4. Strain Prediction Model Based on PSO-BPNN for the Wind Turbine Blade Static Behaviour

### 4.1. Strain Prediction Modeling for the Central of Pressure Side on Edgewise+

When the wind turbine blade bears unilateral tension, the relationship among the strain and the applied loads, loading positions and displacements is nonlinear. The PSO-BPNN methods have outstanding advantages to characterize the nonlinear relationship, thus the strain values of the unmeasured points of the wind turbine blade can be predicted by the PSO-BPNN models. In the PSO-BPNN models for strain predicting, the values of the applied loads, the loading positions and the displacements are used as training inputs, and the strain values are outputted. The 51 sets of strain testing data of the center of the pressure side when loaded on edgewise+ were taken as training samples and five sets of strain testing data were used as test samples, thus a strain predictive PSO-BPNN model for the central of PS was established. The training samples and test samples of the PSO-BPNN model are shown in Table 5 and Table 6, respectively.

**Figure 7:**
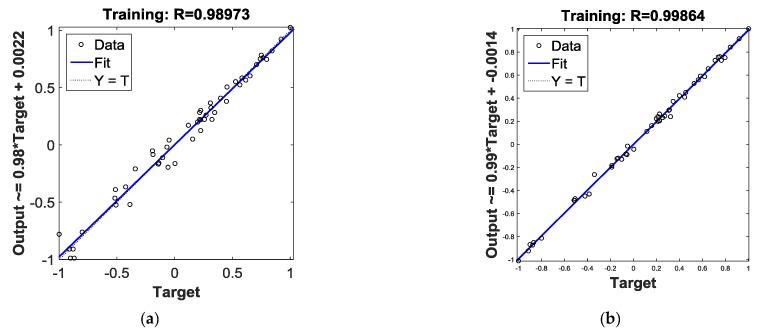

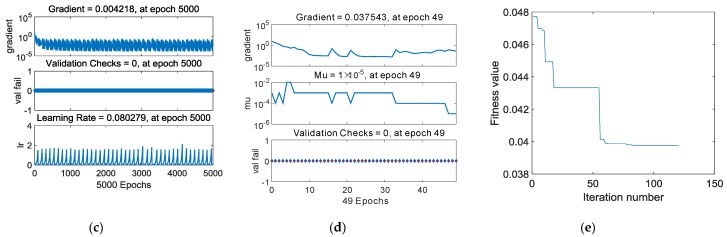
(**a**) Plotting linear regression of BPNN; (**b**) plotting linear regression of PSO-BPNN; (**c**) training state of BPNN; (**d**) training state of PSO-BPNN; (**e**) the Fitness value graph of PSO iteration.

**Figure 8:**
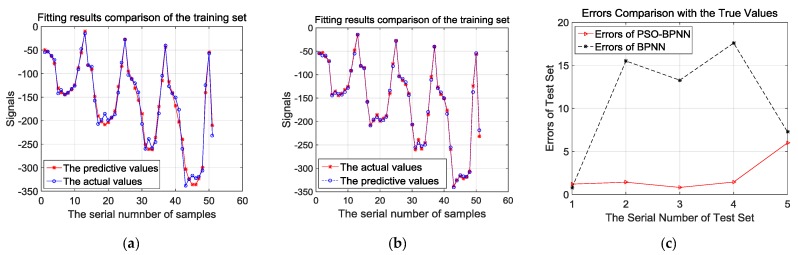
(**a**) Fitting results comparison of the training set by BPNN; (**b**) fitting results comparison of the training set by PSO-BPNN; (**c**) errors comparison of test set by BPNN and PSO-BPNN.

The PSO-BPNN model is operated with the following settings: the maximum number of PSO iterations is 120; both the acceleration constants *c*_1_ and *c*_2_ are set to 2; the maximum particle velocity is 0.8 × *X*_max_ and the minimum particle velocity is 0.6 × *X*_min_; the inertia weight is 0.2. The inputting dimensions are set to 13, and the outputting dimension is set to 1. There are 17 neuron nodes in the hidden layer of the network. The maximum number of network training epochs allowed is 5000; the speed of network learning is 0.1; the minimum convergence error of the training target is set to 0.001. Then, the learning procedure depending on the project samples was calculated. In order to verify the validity and superiority of the PSO-BPNN model, the BPNN algorithm is used solely for the strain predictions to compare with the PSO-BPNN model. The parameters settings of the BPNN algorithm running are shown as follows: The number of inputting dimensions is set to 13 in addition with the number of outputting dimension setting to 1; there are 17 neuron nodes set into the hidden layer, too; the maximum number of network training epochs allowed is set to 5000; the speed of network learning is set to 0.1; the minimum convergence error of the training target is set to 0.001; and the frequency of displaying results is set to every 50 steps. Then, the learning procedure of the project samples was operated. The results of comparisons calculated are illustrated in Figure 7 and Figure 8.

Figure 7a,b plots the linear regression of BPNN and PSO-BPNN respectively. The regression coefficient of PSO-BPNN, measuring the correlation between outputs and targets, is closer to 1 than that of BPNN, which means the PSO-BPNN training has better performance than the BPNN training. Figure 7c,d shows the training state of BPNN and PSO-BPNN, respectively; the PSO-BPNN ran 49 epochs whereas BPNN ran 5000 epochs to the ideal values, so the network trained by PSO-BPNN has higher efficiency than BPNN. In Figure 7e, the fitness values of PSO are shown. When the number of iteration times reaches the early 30s, the fitness values achieve the ideal results in the PSO process.

Figure 8a,b presents the comparison of fitting effects based on the training samples analyzed by BPNN and PSO-BPNN, respectively. They show that the fitting effects of PSO-BPNN are better compared to BPNN. Besides, in order to verify the fitting and predictive abilities of the PSO-BPNN and BPNN algorithms, the result of errors comparison with the true values is shown after the test samples are trained by the two different algorithms in Figure 8c. In Figure 8c, we can see that the PSO-BPNN model has much higher accuracy according to the test results, and its rates of relative errors outputted according to the test sample training are below 6%, while the relative error rates of BPNN models are below 18%, so PSO-BPNN shows a better performance of predictive ability than BPNN.

### 4.2. Strain Prediction Modeling for the Central of Pressure Side on Edgewise−

The methodology is the same as that used in Section 4.1. A strain-predictive PSO-BPNN model for the center of the pressure side loaded on the direction of edgewise− is established. The training samples and test samples of the strain-predictive PSO-BPNN model are shown in Table 7 and Table 8, respectively.

**Figure 9:**
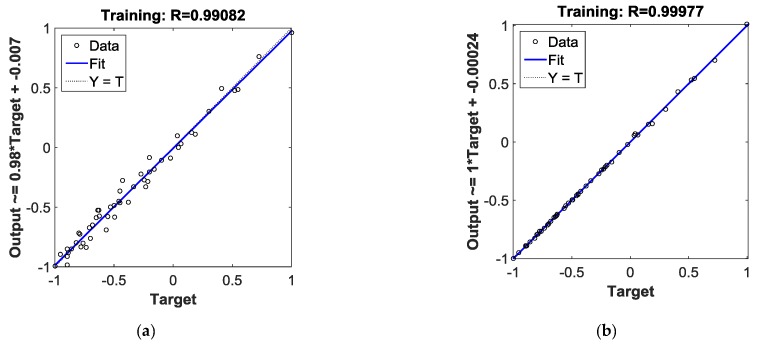

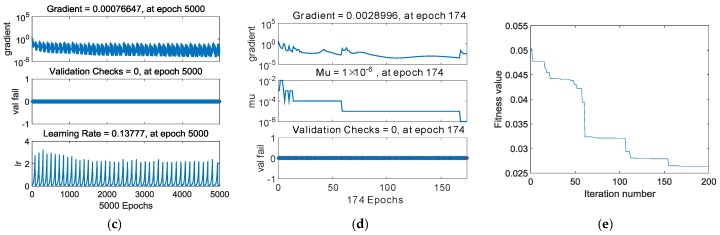
(**a**) Plotting linear regression of BPNN; (**b**) plotting linear regression of PSO-BPNN; (**c**) training state of BPNN; (**d**) training state of PSO-BPNN; (**e**) the Fitness value graph of PSO iteration.

Figure 9a,b shows the linear regressions of BPNN and PSO-BPNN respectively; the regression R-value of the PSO-BPNN model training is 0.99971, which is also closer to 1. It means that the training results of PSO-BPNN show closer correlation with targets than that of BPNN, so the performance of PSO-BPNN training is better than that of BPNN algorithm. Figure 9c,d shows the training state of BPNN and PSO-BPNN, respectively. Figure 9e presents the fitness value graph of PSO. The fitness value levels off between the 165^th^ and the 175^th^ iteration time.

**Figure 10:**
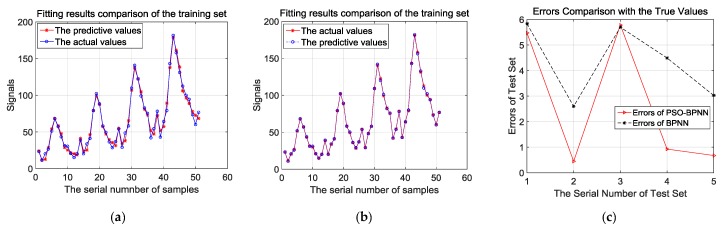
(**a**) Fitting results comparison of the training set by BPNN; (**b**) fitting results comparison of the training set by PSO-BPNN; (**c**) test set errors comparison of BPNN and PSO-BPNN.

According to Figure 10a,b, the fitting figure of the set trained by PSO-BPNN is more precise than that set trained by BPNN. In addition, the test samples are utilized to prove the recognition ability of the trained PSO-BPNN, and the comparison results are shown in Figure 10c. In Figure 10c, we can find that test results of the PSO-BPNN model are much more accurate, the average error rate of PSO-BPNN is less than that of BPNN, and all of the error rates regarding test samples output are below 5.8%, while the relative error rates analyzed by BPNN are all within 6%.

### 4.3. Predicted Results and Verification

In order to demonstrate the effectiveness and feasibility of the proposed PSO-BPNN strain-predictive method, the prediction results based on BPNN methods are used to compare with the FE simulation results. For unmeasured points on the center of the pressure side, 17 points located at 11.00 m, 14.00 m, 16.00 m, 19.00 m, 20.00 m, 20.64 m, 23.00 m, 25.00 m, 33.00 m, 35.00 m, 38.00 m, 41.00 m, 42.00 m, 43.00 m, 46.00 m, 49.00 m, and 52.00 m are chosen to predict their strain value by PSO-BPNN and BPNN methods.

**Figure 11:**
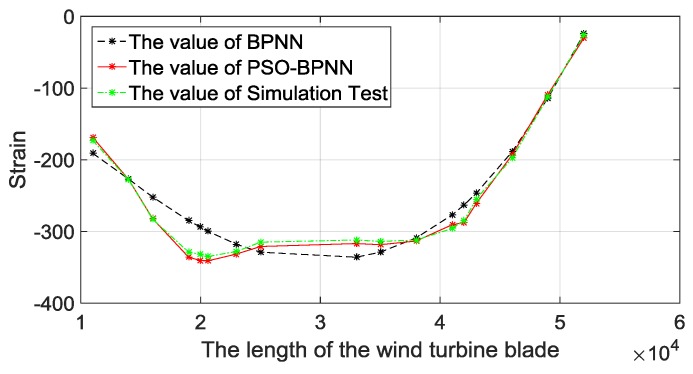
Comparisons of BPNN methods with the FEA.

When loaded on edgewise+, the prediction strain values of the 17 unmeasured points are shown in Figure 11. From Figure 11, we can see that the strain value decreases a lot at the early stage, stays flat for a period of time, and then increases. This result is in accordance with the structural characteristics of blades, owning to a reinforced structure set near the maximum chord length. Besides, the predicted results of all BPNN models are very close to the simulation results, and that means BPNN strain-predictive methods have a high accuracy to predict the strain. Compared with the traditional BPNN method, the PSO-BPNN method has the smallest error, thus the strain-predictive model based on PSO-BPNN is scientific and reasonable.

**Figure 12:**
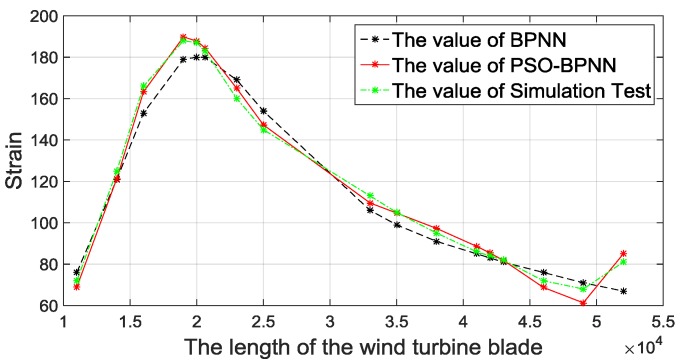
Comparisons of BPNN methods with the FEA.

When loaded on edgewise−, the strain values of the above 17 unpredicted points are shown in Figure 12. The conclusion is the same as the analysis results: When loaded on the direction of edgewise+, all strain-predictive BPNN models have high accuracies, and the PSO-BPNN has the smallest error. 

According to the prediction result of comparisons among BPNN models and the FE simulation results, all BPNN models can predict the strain effectively, while the PSO-BPNN method has higher accuracies compared with traditional BPNN method, and it is more suitable to predict the strain of unmeasured points in the full-scale static testing of wind turbine blades.

## 5. Conclusions

In the full-scale static testing of wind turbine blades, the correlation among the strain and applied loads, loading positions, displacements, etc., is nonlinear, and the number of design variables is numerous, thus the calculation and prediction of the blade strain are very complicated and difficult by traditional numerical methods. Considering these reasons, a strain-predictive PSO-BPNN method is proposed:

(1) Taking the advantages of the BPNN methods in dealing with the nonlinear relationship, a strain-predictive PSO-BPNN model for the full-scale static testing of wind turbine blades was established;
(2) The accuracy of the strain-predictive PSO-BPNN model was verified by comparisons with the traditional BPNN models as well as the ANSYS simulation test. When loaded on the direction of edgewise+, the relative error rate of the strain-predictive PSO-BPNN model is within 6%. Similarly, when loaded on the direction of edgewise−, the relative error rate is also within 6%, which satisfies the blade certification requirements.
(3) The applicability and usability of the strain-predictive BPNN models were verified by comparing with the AYSYS simulation test for 17 unmeasured points. From the comparison results, we can see that all BPNN models have high accuracies to predict strains, and the PSO-BPNN method has the smallest error. Thus, the PSO-BPNN method is much more suitable to predict the strain of unmeasured points in the full-scale static testing of wind turbine blades.

A strain-predictive PSO-BPNN model for full-scale static testing of the wind turbine blade was established in this paper and more strain values can be predicted for unmeasured points in the full-scale static testing of wind turbine blades. This study can provide more data to verify the rationality of blade design and correct the blade defects; the outputs can also be used for life prediction for wind blades, which will be considered as the next work. Moreover, the number of test samples is chosen on the basis of the static test, while the relation between sample number and accuracy will also be considered and studied in the future.

## Figures and Tables

**Table 1:** The main information of the turbine blade.

Rated power	3.0 MW	Maximum chord length	4.32 m
Design life	20 years	Maximum twist angle	15.6°
Blade length	66.5 m	Mass	15,982 kg
Matrix material	Alkali-free glass fibers impregnated with epoxy resin	Reinforcing phase material	Impregnated glass fibre
Main girder stucture	Box beam (2-ply beam)		

**Table 2:** The target load for the five loading positions.

	Distances from the Loading Positions to the Blade Root (m)				
	18.0	30.0	42.0	50.0	60.0
The target load of edgewise+ (kN)	111.5	62.8	45.0	34.5	22.0
The target load of edgewise− (kN)	45.9	81.0	30.0	23.5	24.0

**Table 3:** The applied load of each stage when loaded on edgewise+.

The Load Applications (kN)					
Distances from the Loading Positions to the Blade Root (m)	0	40%	60%	80%	100%
18.00	0	44.92	66.81	89.35	111.95
30.00	0	25.64	37.73	50.68	62.88
42.00	0	18.56	27.87	36.07	45.37
50.00	0	14.05	20.88	27.65	34.53
60.00	0	8.88	13.41	17.74	22.11
66.50	81.14	73.87	70.98	68.20	65.35

**Table 4:** The applied load of each stage when loaded on edgewise−.

The Load Applications (kN)					
Distances from the Loading Position to the Blade Root (m)	0	40%	60%	80%	100%
18.00	0	19.01	27.60	36.79	46.02
30.00	0	32.69	48.94	65.22	81.65
42.00	0	12.28	18.67	24.29	30.49
50.00	0	9.64	14.18	18.97	23.59
60.00	0	9.62	14.50	19.24	23.99

**Table 5:** The training samples used by BPNN and PSO-BPNN model.

**Items**	**Locations of Strain Gauges**	**Load Applications (kN)**					
		***F*_1_**	***F*_2_**	***F*_3_**	***F*_4_**	***F*_5_**	***F*_6_**
1	2000	44.9	25.6	18.6	14.1	8.9	73.9
2	6000	66.8	37.7	27.9	20.9	13.4	71
3	12,000	89.4	50.7	36.1	27.7	17.7	68.2
…	…	…	…	…	…	…	…
50	45,000	112	62.9	45.4	34.5	22.1	65.4
51	33,000	44.9	25.6	18.6	14.1	8.9	73.9
**Items**	**Displacements of the Loading Positions (mm)**					**Strain (με)**	
	***s*_1_**	***s*_2_**	***s*_3_**	***s*_4_**	***s*_5_**	***s*_6_**	***f***
1	88	245	504	742	1093	1284	−55.2
2	135	379	736	1083	1594	1874	−85.4
3	188	507	973	1430	2104	2472	−140.1
…	…	…	…	…	…	…	…
50	240	635	1220	1794	2633	3091	−231.7
51	88	245	504	742	1093	1284	−140.2

**Table 6:** The test samples used by BPNN and PSO-BPNN model.

**Items**	**Locations of Strain Gauges**	**Load Applications (kN)**					
		***F*_1_**	***F*_2_**	***F*_3_**	***F*_4_**	***F*_5_**	***F*_6_**
1	36,000	89.4	50.7	36.1	27.7	17.7	68.2
2	9000	66.8	37.7	27.9	20.9	13.4	71
3	2100	89.4	50.7	36.1	27.7	17.7	68.2
4	12,000	66.8	37.7	27.9	20.9	13.4	71
5	15,000	44.9	25.6	18.6	14.1	8.9	73.9
**Items**	**Displacements of Loading Positions (mm)**						**Strain (με)**
	***s*_1_**	***s*_2_**	***s*_3_**	***s*_4_**	***s*_5_**	***s*_6_**	***f***
1	88	245	504	742	1093	1284	−71.1
2	135	379	736	1083	1594	1874	−91.5
3	188	507	973	1430	2104	2472	−271.2
4	135	379	736	1083	1594	1874	−106.9
5	88	245	504	742	1093	1284	−105.5

**Table 7:** The training samples trained by BPNN and PSO-BPNN model.

**Items**	**Locations of Strain Gauges**	**Load Applications** **(kN)**					
		***F*_1_**	***F*_2_**	***F*_3_**	***F*_4_**	***F*_5_**	
1	6000	19	32.7	12.3	9.6	9.6	
2	12,000	27.6	48.9	18.7	14.2	14.5	
3	21,000	36.8	65.2	24.3	19	19.2	
…	…	…	…	…	…	…	
…	…	…	…	…	…	…	
51	51,000	46	81.7	30.5	23.6	24	
**Items**	**Displacements of the Loading Positions (mm)**						**Strain (με)**
	***s*_1_**	***s*_2_**	***s*_3_**	***s*_4_**	***s*_5_**	***s*_6_**	***f***
1	66	198	419	616	912	1079	11.3
2	103	307	629	924	1368	1618	40.9
3	141	417	838	1230	1822	2152	140.8
…	…	…	…	…	…	…	…
…	…	…	…	…	…	…	…
51	181	528	1054	1544	2286	2697	76.9

**Table 8:** The test samples trained by BPNN and PSO-BPNN model.

**Items**	**Locations of Strain Gauges**		**Load Applications (kN)**				
		***F*_1_**	***F*_2_**	***F*_3_**		***F*_4_**	***F*_5_**
1	33,000	19	32.7	12.3		9.6	9.6
2	36,000	27.6	48.9	18.7		14.2	14.5
3	27,000	27.6	48.9	18.7		14.2	14.5
4	39,000	36.8	65.2	24.3		19	19.2
5	45,000	36.8	65.2	24.3		19	19.2
**Items**	**Displacements of the Loading Positions (mm)**						**Strain (με)**
	***s*_1_**	***s*_2_**	***s*_3_**	***s*_4_**	***s*_5_**	***s*_6_**	***f***
1	66	198	419	616	912	1079	34.6
2	103	307	629	924	1368	1618	52.9
3	103	307	629	924	1368	1618	70.5
4	141	417	838	1230	1822	2152	70.2
5	141	417	838	1230	1822	2152	54.9

## Abbreviations

BPNN	Back Propagation Neural Network
PSO	Particle Swarm Optimization
PSO-BPNN	Back Propagation Neural Network Improved by Particle Swarm Optimization
PS	Pressure Side
SS	Suction Side
